# Single-incision laparoscopic surgery - current status and controversies

**DOI:** 10.4103/0972-9941.72360

**Published:** 2011

**Authors:** Prashanth P Rao, Pradeep P Rao, Sonali Bhagwat

**Affiliations:** Department of Minimally Invasive Surgery, Mamata Hospital, Dombivli, Mumbai, India; 1 Department of Surgery, BYL Nair Hospital and TN Medical College, Mumbai, India

**Keywords:** E-NOTES, LESS, single-incision laparoscopy, single-port access

## Abstract

Scarless surgery is the Holy Grail of surgery and the very raison d’etre of Minimal Access Surgery was the reduction of scars and thereby pain and suffering of the patients. The work of Muhe and Mouret in the late 80s, paved the way for mainstream laparoscopic procedures and it rapidly became the method of choice for many intra-abdominal procedures. Single-incision laparoscopic surgery is a very exciting new modality in the field of minimal access surgery which works for further reducing the scars of standard laparoscopy and towards scarless surgery. Natural orifice translumenal endoscopic surgery (NOTES) was developed for scarless surgery, but did not gain popularity due to a variety of reasons. NOTES stands for natural orifice translumenal endoscopic surgery, a term coined by a consortium in 2005. NOTES remains a research technique with only a few clinical cases having been reported. The lack of success of NOTES seems to have spurred on the interest in single-incision laparoscopy as an eminently doable technique in the present with minimum visible scarring, rendering a ‘scarless’ effect. Laparo-endoscopic single-site surgery (LESS) is, a term coined by a multidisciplinary consortium in 2008 for single-incision laparoscopic surgery. These are complementary technologies with similar difficulties of access, lack of triangulation and inadequate instrumentation as of date. LESS seems to offer an advantage to surgeons with its familiar field of view and instruments similar to those used in conventional laparoscopy. LESS remains a evolving special technique used successfully in many a centre, but with a significant way to go before it becomes mainstream. It currently stands between standard laparoscopy and NOTES in the armamentarium of minimal access surgery. This article outlines the development of LESS giving an overview of all the techniques and devices available and likely to be available in the future.

## RAISON D’ETRE OF LESS-NOTES AND ITS INHERENT PROBLEMS

Since the first laparoscopic cholecystectomy was described by Muhe in 1985, and later published by Mouret, Perissat and Dubois in 1987 and 1988,[1–3] laparoscopic surgery has expanded in leaps and bounds to become the standard procedure for many intra-abdominal surgeries. The quest for scar reduction beyond standard laparoscopy led to the experimentation with natural orifice surgery. The first description of the procedure to be known as natural orifice translumenal endoscopic surgery (NOTES) is credited to Kalloo *et al*. in 2000, where they demonstrated the feasibility of a per-oral transgastric endoscopic approach to the peritoneal cavity with long-term survival in animals.[4] Gettman and colleagues in 2002 reported their series of transvaginal porcine nephrectomies.[5] Drs Rao and Reddy reported the first human case of NOTES in 2004 with a transgastric appendectomy.[6] In July 2005, there was a meeting of some of the leaders of the American Society of Gastrointestinal Endoscopy (ASGE) and the Society of American Gastrointestinal and Endoscopic Surgeons (SAGES). The deliberations of this group thence called the Natural Orifice Surgery Consortium for Assessment and Research (NOSCAR) group, were published as a White Paper of the ASGE/SAGES working group on NOTES.[7] The paper recommended and emphasised the need for Institutional Review Board (IRB) approval before doing any human cases. ‘Pure’ NOTES is that which is only performed through natural orifices. This could be trangastric or transoesophageal[8] (per oral access), transvaginal, transcolonic[9] or transvesical.[10] Rendezvous NOTES has been used to describe an approach where more than one portal of entry is used to avail of triangulation and hybrid NOTES is used to describe any natural orifice surgery when an additional laparoscopic port is used transabdominally.[11–14] *Robotic NOTES* is an exciting new development using the Da Vinci surgical Robot (Intuitive Surgical, Sunnyvale, CA) in animals to perform various reconstructive and ablative procedures.[15,16] The *Transvaginal route* has been used with some amount of success for NOTES, most commonly used in clinical practice for appendectomies and cholecystectomies and some urological applications.[13,17]

Gastrointestinal endoscopists are most familiar with the transgastric route and they have driven the innovation in this field. The primary difficulties associated with this route seem to be the difficult orientation after retroflexing the scope in the peritoneal cavity, particularly for a cholecystectomy or upper abdominal procedures, lack of a device for secure closure of the stomach or colon,[18] as well as inadequate amount of light to illuminate the capacious peritoneal cavity.

The vaginal route has had the most success as closure of the vagina is fairly easy and being expansile, it is possible to use rigid laparoscopic instruments which surgeons are more familiar with. Obviously this can be performed in only a small subset of the population. Some gynaecologists remain concerned about pelvic adhesions and subsequent infertility after these procedures. There are also concerns about spread of endometriosis and that this also may lead to dyspareunia in the postoperative period.[19] Besides being limited to the fairer sex, it may not be fair to use it in the younger population who have not completed child bearing and may be even difficult and unethical to use it in the sexually inactive young patients. This would than limit it to the subset of multiparous middle-aged women.

Furthermore, the scopes being used for NOTES are the standard off the shelf double channel endoscopes. As two instruments are passed down the channel of the endoscope there is considerable limitations in their use today.[6] Firstly, it is difficult to achieve triangulation as the channels are parallel. Secondly, the flimsy endoscopic instruments are hard pressed to hold and retract normal organs, let alone organs inflamed by disease and inflammation. Thirdly, use of the flexible endoscope to pass these instruments has the disadvantage of excessive mobility making these procedures more cumbersome as the endoscope easily gets lost in the insufflated and capacious abdominal cavity. Other than the difficulties encountered with NOTES, the very act of causing a perforation in an otherwise normal organ to get at a pathological organ seems to defy logic and common sense and may be detrimental and at times disastrous, whenever the closure of the stomach or colon fails or is insecure.

NOTES is an exciting new technology but has a long way to go before it can be used in routine clinical practice. This led surgeons to look towards better avenues to reduce/eliminate scars beyond standard laparoscopy. Development of new multichannel access devices and articulating instruments would lead to emergence of single-incision laparoscopic surgery. This would eventually be brought under the term laparo-endoscopic single-site surgery (LESS).[20] LESS managed to fill the present void left by the lack of applicability of NOTES in the immediate future.

### Single-incision laparosocpic surgery

Single-port laparoscopy is not new. It had been around for more than 30 years. The gynaecologists were doing tubal ligation with a single-puncture laparoscope since the late 70s.[21,22] This technique works well for gynaecological surgery as the uterus can be manipulated from below. These early instruments had offset eyepieces with a straight operating channel through which an applicator for the silicone ring to occlude the tubes, could be passed.[23,24] Vaginal manipulation of the uterus obviated the need for retraction. The advantages of using a second instrument with triangulation were noted at the time.[25] Pelosi *et al*. even reported the performance of advanced pelvic extirpative surgery using the single puncture.[26] Appendicectomies have been done with a single puncture as early as 1992.[27] In this technique the appendix is coaxed out of the umbilicus to complete the task after caecal mobilisation.[28] More recently this has even been described with transumbilical flexible endoscopy.[29] The use of multiple trocars rapidly gained popularity over the disadvantages of a single puncture. As conventional laparoscopy became popular even for complex procedures in surgery, it was usually carried out through four or more ports. Increasing the number of ports led to reduced cosmesis, more pain and increased risk of complications due to port site infections and hernias.[30,31] One advantage of reducing the number of ports over cosmesis, would be to reduce these complications. The minimal access surgical techniques have come a full circle with the single-incision surgery gaining popularity once again. Furthermore, single-port/single-site surgery may be a closer step towards that elusive goal of NOTES.[32]

## TECHNIQUES OF LESS

### The single-incision but multi-port technique

**Figure 1:**
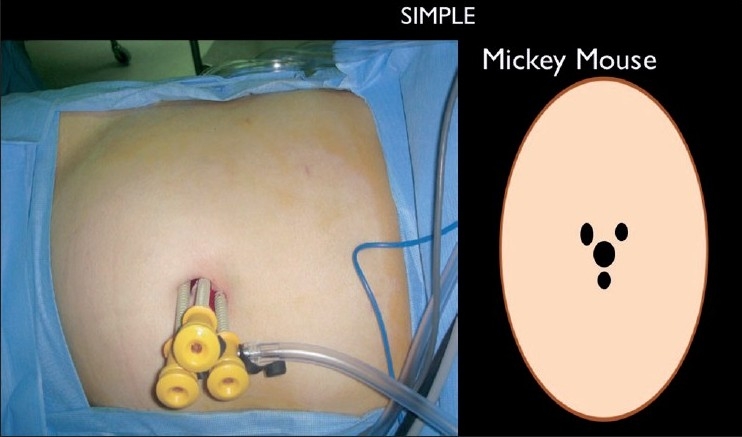
‘Mickey Mouse’ configuration of ports in SIMPLE.

This has been aptly described as SIMPLE by some authors. The transumbilical technique for cholecystectomy, without additional incisions, was described first by Navarre *et al*. in 1997 and later Piskun *et al*. in 1999,[33,34] but failed to gain popularity due to lack of proper instrumentation. They used sutures to retract the gallbladder. Cuesta *et al*. published this as the ‘invisible cholecystectomy’ in 2008 and used Kirschner wires to anchor the gall bladder.[35] This was further refined and popularised by Curcillo and King in 2007 and published subsequently with their original technique of entry.[36,37] In their technique, a skin incision at the umbilicus, allows a flap of the umbilicus to be raised, allowing for three to four separate sheath incisions at distances of 2-3 cms from each other in the ‘Mickey Mouse’ (after the shape of the famous Walt Disney character) configuration [Figure 1]. This makes it possible to insert three or even four low profile trocars which can permit one optic and two working instruments and the possibility of an additional grasping instrument as well. Around the same time, Cadeddu and associates were working on a similar technique, using three separate trocars through a single-incision in the umbilicus to do nephrectomies.[38] When low profile and short trocars were used in this technique, they would reduce the clashing of the instruments close to the abdominal wall. The understanding was that going through separate fascial defects at a slight distance from each other increased the maneuverability of the working ports and negated gas leakage. The obvious disadvantage was that the Swiss cheese defects left behind could be difficult to close securely.

### Use of a singular access device

**Figure 2:**
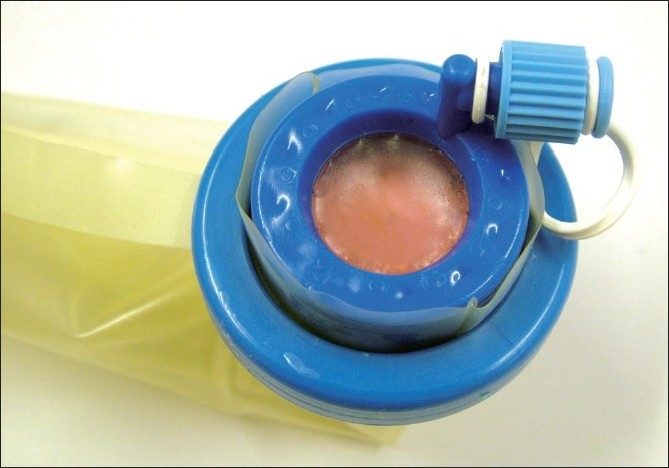
The original R-Port.

**Figure 3:**
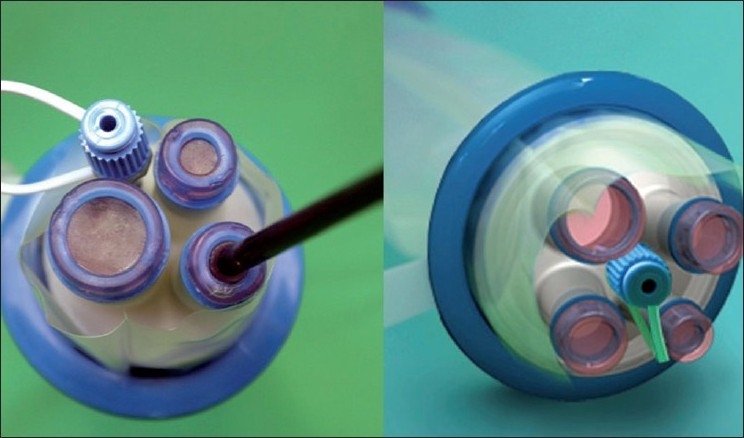
Tri-port and Quad port.

**Figure 4:**
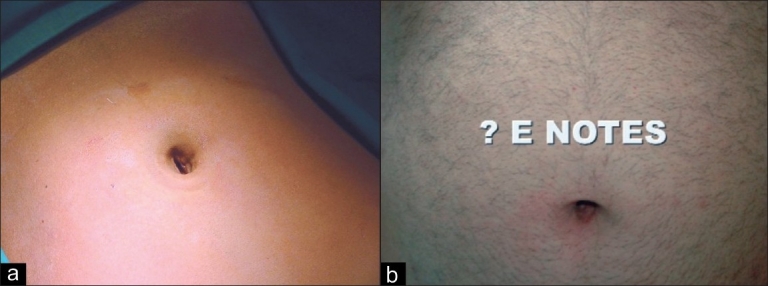
(a) Umbilical incision (b) Immediate post op and 3 weeks after.

**Figure 5:**
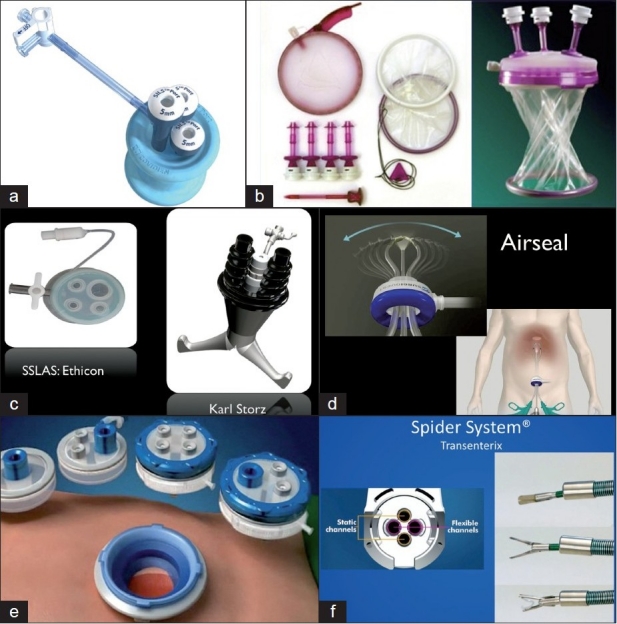
a) SILS Port (TM), b) Gelport, c) SSLAS and X-Cone d) Airseal, e) Octoport and f) Spider system

**Figure 6:**
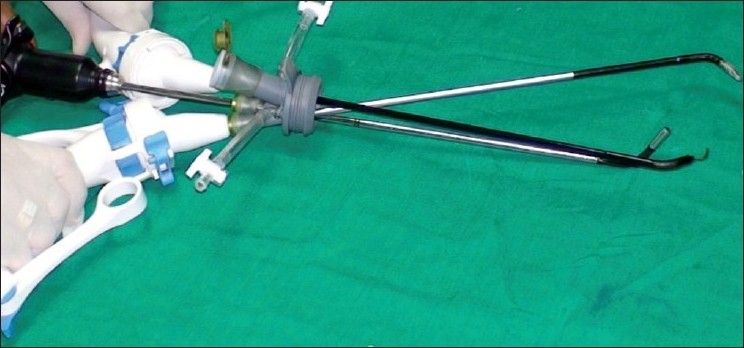
Articulating instrument.

**Figure 7:**
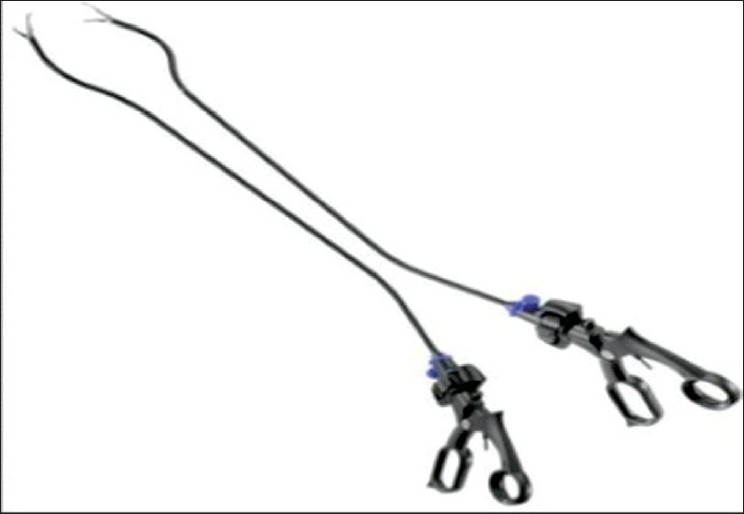
Prebent instruments.

The second technique involves the use of a singular access device that permits the ingress of 3 or 4 instruments through a single opening in the umbilicus into which the device is first inserted. The first of these devices to be available was the R- Port^™^ (Advanced Surgical Concepts, Wicklow, United Kingdom). The first cases of single-port access using an access device in the form of the prototype of the R-Port were done by Rao *et al*. in general surgery and urology in May 2007 and were reported at the World Congress of Endourology in 2007 and Asia Pacific Congress of the Endoscopic and Laparoscopic Society of Asia (ELSA) in 2007 and also later published.[39–41] The authors managed 85% of their cholecystectomy cases in the pilot series without any additional instrumentation and had to use extra needles for retraction in another 10%. One case required an additional port. The first R-Port [Figure 2] was just a single-gel interphase that could be perforated many times to get the instruments in. However, sometimes the holes would coalesce and cause gas leakage. This was later modified to the multi-valve ports available today i.e. the Tri-Port and the Quad-Port [Figure 3]. Desai *et al*. used the R-Port for reconstructive as well as ablative procedures through the umbilicus in clinical cases giving almost a scarless appearance after surgery.[42] The umbilicus being an ubiquitous cicatrix from birth could be used to conceal the access into the abdominal cavity as shown by these pioneers [Figure 4, 4a] The early experience with LESS could be attributed to the development of new access devices [the R-Port^™^ and the Uni-X^™^] and new articulating instruments [Real Hand^™^ by Novare Surgicals, Cupertino, CA]. The initial reports paved the way for a flood of access devices to enter the market [SILS port^™^ by Covidien, SLASS^™^ by Ethicon, Air Seal^™^ by Surgiquest, Octoport^™^, Daikin Surgical, Korea and X-Cone^™^ by Karl Storz] [Figure 5a–e] The Spider^™^, Transenterix system also had unique flexible arms [Figure 5f]. The GelPort^™^, Applied Medical, USA was already in use as a hand port and was now marketed as an access device as well. These new access devices permitted the surgeon to insert more than two instruments and an optic, with trocars as in the GelPort and the SILS^™^ port and without as in the R-Port^™^, through the same port.[39–41,43] The articulating instruments [Figure 6] or prebent [Figure 7] instruments could be used through these access devices and give a sense of triangulation.[38,44] These ports were inserted through an incision measuring anywhere between 17 and 50 mm depending on the port and the organ to be accessed/removed. The average incision for a cholecystectomy was about 20-25 mm. It made sense to take slightly bigger incisions and get more play between the instruments when the organ to be removed was larger as in the case of radical or donor nephrectomy or a colectomy. The plastic sheath of these ports doubled up as a wound protector in most but not all cases.

### Terminology of single-port surgery

In the initial reports of single-port surgery, every paper was published using a different nomenclature for the surgery. Some of the terms used to describe this surgery included SPA (single-port access), SLaPP (single laparoscopic port procedure), SILS (single-incision laparoscopic surgery), OPUS (one-port umbilical surgery), SIMPLE (single-incision multi-port laparo endoscopic surgery), NOTUS (natural orifice transumbilical surgery) and E-NOTES (embryonic natural orifice transumbilical endoscopic surgery, as the umbilicus is an embryonic structure) among others [Table 1].[36,38,40,44–48] There was a need to unify these surgeries under a single nomenclature, so that it would be easier to document new developments and guide this emerging technique. A multidisciplinary consortium of surgeons met at the Cleveland Clinic in July 2008 (LESSCAR- Laparo-Endoscopic Single Site Surgery Consortium for Assessment and Research), which brought out a white paper on this subject. They suggested the name of LESS Surgery for all such procedures which used a single site for access. The consortium also suggested a standardization for reporting these surgeries as follows: “To clearly and fully convey all procedural details, a ‘mandatory descriptive second line’ in all scientific publications, which provides all relevant information at the very outset, such as: length and location of incision (abdominal [umbilical or extra-umbilical], thoracic, or pelvic); approach (transperitoneal, retroperitoneal, percutaneous intraluminal, transluminal); number/type of ports used; laparoscopic, endoscopic or robotic; type of optics used (rigid, flexible, coaxial cable, chip on tip, etc); type of instruments used (straight, curved, articulating or flexible); and whether any ancilliary 2-mm needlescopic instrumentation or extra ports employed.”[20] Similar to NOSCAR, the mandate for LESSCAR included creating a multidisciplinary group, solicit funding, establishing a registry and database of procedures, encouraging and guiding LESS research and collaborating with established professional organizations to facilitate LESS presentations at their meetings, among other things. LESSCAR membership was kept inclusive, but insisted on IRB approval to carry out LESS research.[20]

**Table 1:** Acronyms used to describe single-port/single-site surgery

SPA - Single-port access
SILS - Single-incision laparoscopic surgery
OPUS - One-port umbilical surgery
E-NOTES - Embryonic natural orifice transumbilical endoscopic surgery
SIMPLE - Single-incision multi-port laparo-endoscopic surgery
SPS - Single-port surgery
VSUS - Visibly scarless urological surgery
SIL - Single-incision laparoscopy
SPL - Single-port laparoscopy
R-NOTES - Robotic-assisted natural orifice transumbilical endoscopic surgery
U-NOTES - Umbilical natural orifice transluminal endoscopic surgery
LESS - Laparo-endoscopic single-site surgery
SLaPP- Single laparoscopic port procedure
NOTUS- Natural orifice transumbilical surgery
SLiPP- Single laparoscopic incision and port procedure

### Problems with LESS and instrumentation

As with most new surgical techniques, the early development of LESS surgery was fraught with problems. The main problems with performing LESS surgery are a loss of triangulation, clashing of instruments and the instruments with the telescope and camera head, and a lack of maneuverability. There was also the additional problem of decreased exposure at the hepatico-cystic triangle in a cholecystectomy. This new technique of surgery could progress only by circumventing these issues [Table 2]. LESS has mainly been driven by availability of new technologies which have enabled surgeons to overcome these difficulties.

**Table 2:** Tackling problems in single-site surgery

Problem	Solution
Damage to light fiber of conventional laparoscope	Use optic with coaxial light fiber (e.g. EndoEye (TM))
Clashing of telescope with instruments	Use of deflectable tip telescope
Loss of triangulation	Use of articulating or prebent instruments
Clashing of trocars within the abdominal cavity and outside	Use of low profile trocars/short trocars
Multiple incisions unsightly, worry about sheath closure, herniation	Use of access device with single-incision (Triport, Quadport, SILS port, XCone)
Clashing of camera head with instruments	Use optic with chip on tip [e.g. EndoEye (TM)], use long telescope, make assistant sit, hands in a different plane
Lack of exposure	‘Puppetry’ traction on gall bladder using sutures intra or extracorporeal, use of Endograb (TM)
Difficulty in movements of instruments	Slightly larger incision like that of 22-25 mm instead of 17-mm improves play
Inability to insert 10-mm clips	Special clip applier with 10-mm jaws and 5-mm shaft (gel valves), bigger incision
Access device slips out/leaks gas	Sheath incision too big, suture one end

**Figure 8:**
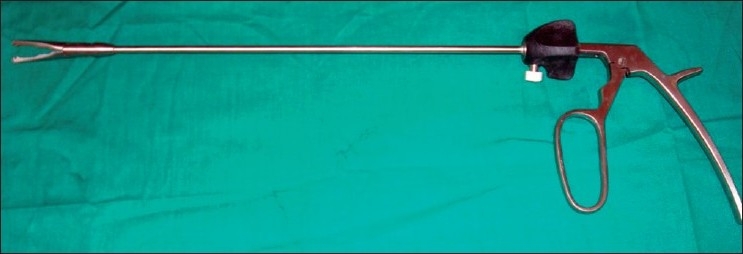
Clip applier with 10-mm jaws and 5-mm shaft.

The early experience with LESS could be attributed to the development of new access devices and new prebent or articulating instruments developed by the pioneers. These new access devices permitted the surgeon to insert more than one instrument and an optic through the same port.[39–41,43] The articulating instruments could be used through conventional straight trocars which were passed through a single site (usually the umbilicus) and give a sense of triangulation.[38,44] Some special instruments developed for the purpose included a clip applier with 10-mm jaws and a 5-mm shaft which could be inserted through a gel valve of the R- Port. This allowed insertion of the jaws with only one instrument in place and then reinsertion of the grasper. This meant that one could accommodate a 10-mm clip through a 17-mm incision which would normally permit only three 5-mm instruments.[41] [Figure 8].

**Figure 9:**
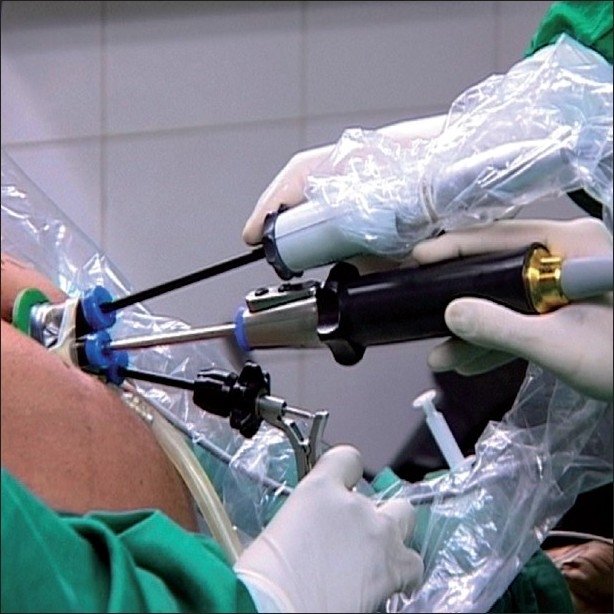
Swordfighting or chopsticks effect.

**Figure 10:**
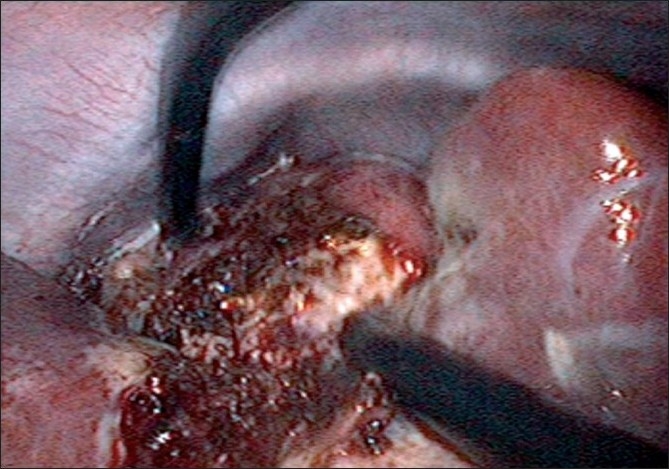
Pseudotriangulation.

**Figure 11:**
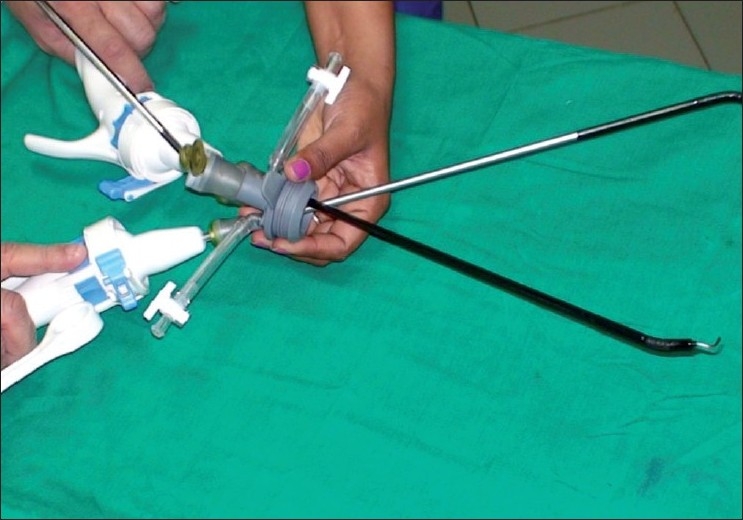
Cross-triangulation.

**Figure 12:**
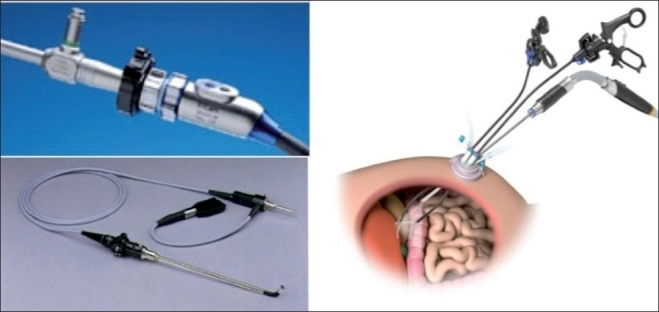
Endo Eye (TM) with deflectable tip and coaxial cable.

Fortunately, technology in optics and instrumentation was keeping stride. Triangulation is one of the fundamental concepts of laparoscopic surgery, as it permits traction on tissues to facilitate dissection along normal anatomical planes. One of the main problems that single-port surgery brought along with lack of triangulation was ‘swordfighting’ or the ‘chopsticks’ effect [Figure 9] as the instruments going in close to each other clashed with one another and the camera head and telescope. Articulating instrumentation allows for triangulation or at least the effect (pseudotriangulation?) [Figure 10] to occur intracorporeally despite the entry points being adjacent to one another through the same skin incision.[38] A few novel ideas like prebent instruments were developed with this type of surgery. Prebent instruments cannot be passed through conventional trocars which are straight and rigid.[39] They can, however, be passed through some of the newer generation of access devices like the Triport^™^ and Quadport^™^, which have a very low profile inside and outside the abdominal wall. Articulating instruments were originally developed to mimic the freedom of movement afforded by the robotic wrist of the DaVinci surgical robot (Intuitive Surgical, Sunnyvale, CA). They could be used through conventional straight trocars as used by the robotic arms. They were suitable for both types of LESS. Articulating graspers, Endoshears (Autosuture, Norwalk, CT) and needle holders were available. Roticulating instruments (Covidien, Dublin, Ireland) have a 0-80^°^ range of motion, allowing infinite freedom for tip adjustment. They have a spin lock mechanism that allows them to use it as a rigid instrument. Handheld Autonomy Laparo-Angle Instruments (Cambridge Endo, Framingham, MA) have been designed to simulate the surgeons hand in motion, and with its axial rotation knob and exclusive angle locking mechanism, provide a better control. The disadvantage is that using all these articulating instruments has a significant learning curve before one handles them dexterously. All these difficulties may result in crossing of instruments at times, or ‘cross-triangulation’, a maneuver frequently used in single-incision surgery, though it was frowned upon in open surgery [Figure 11]. As one gets familiarised with this surgery it is easy to see that an instrument passed from the left end of the port automatically gravitates to the right of the organ and vice versa. The telescope was the other problem as the camera head and telescope clashed with the instruments. This could be circumvented by using long or specific telescopes. The Endo Eye (Olympus, Tokyo, Japan) was suited for this as it came with the ‘chip-on-tip’ technology which meant that it had a streamlined profile with a single coaxial cable and this reduced the cluttering and clashing with the bulky camera head. It also came with a deflectable tip [Figure 12]. Also, as initially demonstrated by Raman *et al*., in the same publication, the use of a deflectable tip laparoscope is uniquely suited to this sort of surgery. It must however be noted that use of this type of laparoscope does have a learning curve for the assistant holding the scope. Alternately as the surgeon is doing one handed dissection, he / she could choose to hold the scope himself/ herself. The problem of the perpendicular light cable of regular telescopes and the bulky camera heads clashing with the instruments can be done away with by the use of a telescope with a coaxial light cable or simply a longer scope so that the camera head moves away from the surgeon’s hands. Some surgeons prefer the assistant to sit so that the assistant’s hands move in a different plane, reducing the clashing.

In addition to use of the new devices, single-site surgery was also carried out using access devices which were already available along with regular laparoscopic hand instruments.[49] Some of the earlier hand access devices like the Gelport^™^ are suited to a single-incision surgery allowing multiple trocars/instruments to be passed through them. They have the additional benefit of allowing for removal of the specimen easily after ablative surgeries for larger organs.

**Figure 13:**
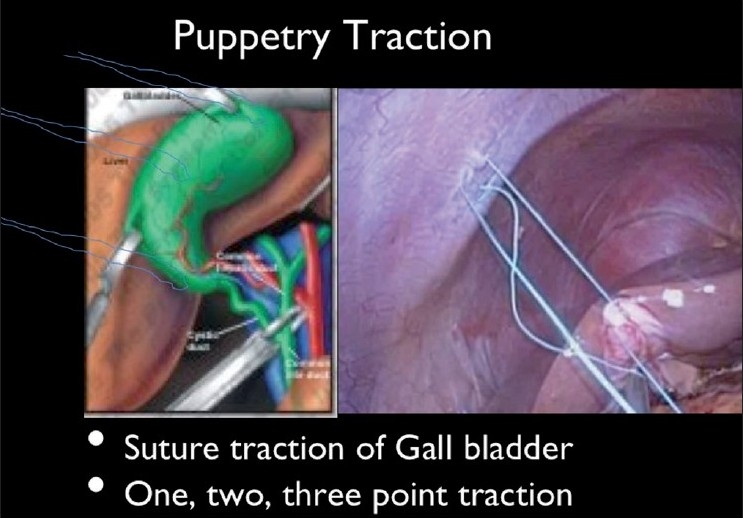
Puppetry traction with sutures.

**Figure 14:**
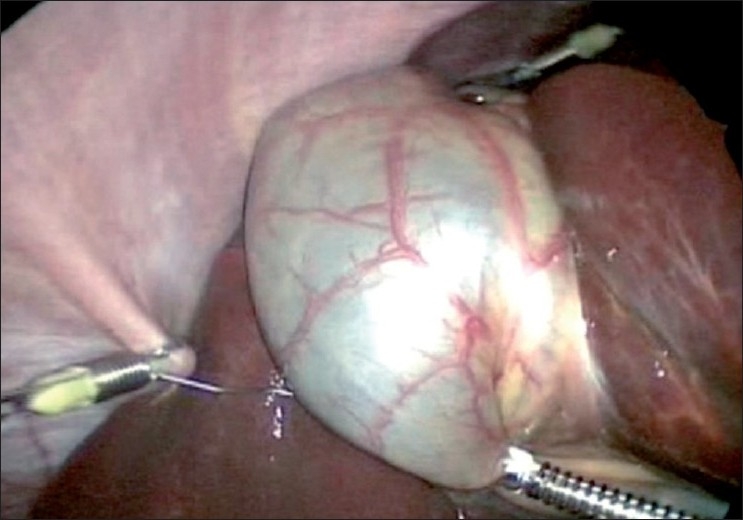
Endo grab (TM).

There are other concerns with LESS. The larger incisions and raising a flap at the umbilicus may lead to theoretically, more wound problems and increased rate of incisional hernias, though a 2-year follow-up has failed to list such adverse effects in literature. It is, however, important to close these fascial defects as one would any laparotomy scar. There are worries that lack of proper retraction at the Calot’s or the hepatico-cystic triangle may lead to a reemergence of increased bile duct injuries as seen in the early days of laparoscopic cholecystectomy. Various methods and instruments have been devised to circumvent the issue of retraction. Retraction of the gallbladder can be done in many ways- ‘Puppetry’ traction technique uses single or multiple sutures either taken extracorporeally or intracorporeally, harnessing the fundus and Hartmanns pouch to the abdominal wall [Figure 13]. An Endograb^™^ (Virtual-Ports, Israel) hook has been devised that is used to anchor the gall bladder to the parietes to get better exposure of the Hepatico-cystic triangle to make up for the lack of a retracting instrument [Figure 14].

Urologists took to LESS must faster than general surgeons and it was rapidly used for a lot of advanced urological procedures.[45] It has also been used widely in gynaecological applications.[50] Unfortunately, like in standard laparoscopic surgery, LESS is most commonly used in general surgery for the cholecystectomy and appendectomy. There are at least three series published of more than a 100 cases of laparoscopic cholecystectomy including a multi-institutional review, and the largest 2-year follow-up by Curcillo *et al*. Erbella *et al*. had a 98% success rate with two converted/rescued to standard laparoscopy. Rivas *et al*. managed to do 87% of their cases with two trocars in the umbilicus and needed an additional port for the other cases. There have been no major complications or hernias reported in these large series.[51–54] There are a few centres which have reported other surgeries like colectomies, splenectomies, fundoplication, hernias, adrenalectomies, with seemingly good results. Magnetic retractors, sutures and slings have also been used to facilitate these surgeries.[55–59]

### LESS-the future

Since the introduction of single-port access in 2007, it has rapidly gained popularity with surgeons as well as the industry. Several hundred cases have been performed with a significant number being reported in peer-reviewed journals [Table 3].[60,61] With LESS, the surgeon has a more conventional view of the field of surgery as compared with a transvaginal or transgastric view obtained in NOTES. The equipment used for LESS is familiar to surgeons already doing laparoscopic surgery. Most importantly, it is easy to convert LESS to conventional laparoscopy by adding a few trocars or even needles or 2-mm needlescopic instruments. Single-port purists prefer the term ‘single-port rescue’ for such procedures rather than ‘conversion’, a term that has denoted transformation to open surgery from conventional laparoscopy. ‘Reduced port surgery’ is another term used to denote performance of these laparoscopic procedures through anything less than the standard number of ports used or if additional ports are used/needed in LESS.[62] This ensures the safety of the patient during the surgeons early experience and learning curve with this sort of surgery.[63] Most of the reported LESS procedures seem to have equivalent efficacy to conventional laparoscopy including operative times, blood loss and length of hospital stay. More importantly the complications like bile duct injuries or incisional hernias have not seen a rise as in the initial years of laparoscopy. The acceptance among patients is very high when told that one incision will be used instead of four or five. What remains to be seen with time and increasing numbers, which will lend statistical validity to the comparisons, is whether LESS will offer enough advantages (cosmetic or otherwise) to replace conventional laparoscopy. Other benefits like pain relief, if any, may prove more difficult to objectify. The only randomised study carried out between transumbilical surgery and standard laparoscopic cholecystectomy showed improved pain scales.[64] There is a significantly longer learning curve with LESS beyond conventional laparoscopy and the benefits need to outweigh this. There are already comparisons being performed in surgeries such as donor nephrectomies.[65,66] This is where LESS can make a significant contribution by making it less cumbersome and more acceptable to healthy people who have to donate organs. The Da Vinci Robotic system (Intuitive Surgical, Sunnyvale, CA) has been used with some success in single-incision surgery. The Robotic Endowrist technology with three dimensional visualization makes it attractive for reducing the technical challenges posed by single-site surgery.[67]

**Table 3:** Landmark series of single-incision laparoscopic surgery

Author	Year	N	Conver.	Compli.	Time	Type of LESS	Comment
Piskun	1998	10	0	0	NR	Transumbilical Multi-port	Used Navarra technique
Cuesta	2008	10	0	0	70	Multi-port	Kirschner wires
Navarra	2008	30	0	0	123	Two-port	First using transumbilical two port
Rao	2008	20	3(15%)	0	40	R-Port	First using access device
Palanivelu	2008	10	4(40%)	1(10%)	148	Multi-port	Used flexible endoscope for appendectomy
Bucher	2009	11	0	0	52	Access device	Also series on colectomies
Podolsky	2009	5	0	0	121	Multi-port ‘MickeyMouse’	Curcillo technique SPA/SIMPLE
Tacchino	2009	12	0	0	50	Three 5-mm trocars	Roticulating endoshears
Kuon Lee	2009	37	5(13.5)	2(5.4)	83	Assembled access device	Mesentric and R Hepatic duct inury
Zhu	2009	26	0	0	62	3-5-mm transumbilical trocars	5-cm longer instruments
Rivas	2009	100	13%	0	50.8	SILS Port	‘Puppetry’
White	2009	100	6%	4%	119	Access device	Urology cases 11-month FU
Erbella	2010	100	2%	0	NR	SIMPLE	6-month F U
Curcillo	2010	297	8.7%	minor	71	All types	Multi-institutional

N, number of patients; Conver., conversions, Compli., complications, NR, not recorded; LESS, Laparo-endoscopic single-site surgery

**Figure 15:**
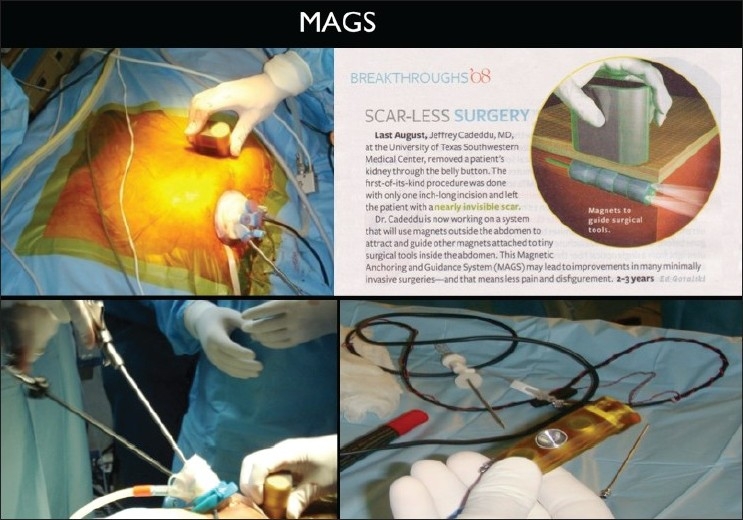
Magnetic anchoring and guidance system technology.

Another exciting technology being brought out is the MAGS or the magnetic anchoring and guidance system. In this a magnetic camera is placed in the abdominal cavity through the umbilical incision taken to insert the single-port access device. The magnetic camera is then controlled by a stack of magnets placed on the abdominal wall, which are used to move it to the organ of interest [Figure 15]. The camera transmits the image via a wire which exits the abdomen through the single-port device or by the side of it. Caddedu and Rao did the first clinical human cases using the prototype magnetic camera for a single-port nephrectomy and appendectomy, respectively, which was presented at the SAGES 2009 meeting and this was later published.[68] The obvious advantages of MAGS for single-port surgery are that it leaves one more portal of the access device free to house another instrument. Current limitations include inability to clean the lens, a cumbersome wire that has to exit the abdomen, insufficient lighting and a magnetic strength that can only accommodate a thin abdominal wall. There is also the danger of a magnetic footprint if left in place for a long time. Future cameras could be wireless, have lens cleaning systems and better lighting and magnetic controls. This MAGS technology could be also extrapolated to magnetic retractors, hooks and dissecting instruments in the future.[69]

## CONCLUSIONS

Single-port surgery has left its mark in minimal access surgery and has been adopted by some centres with very good results for all kinds of intra abdominal surgeries. All the initial studies show it to be feasible, reasonably safe and cosmetically advantageous to standard laparoscopy. Obviously one would not see a stark benefit as one did between open surgery and laparoscopy when it first began. It will no doubt be spurred on by rapid advances in technology and better instrumentation that is likely to follow. Experienced laparoscopic skills are obviously needed to accomplish safe single-port surgery. The cost factor, given the access devices and other instrumentation, is significantly more as are the learning curve and operative times. Of course, the cost would be negated if one used the SIMPLE technique and standard laparoscopic instruments, but the other problems remain. Open surgery had a wide incision that accommodated the surgeon’s hands. Laparoscopy with its tunnel vision took away the space for the hands but added triangulation to make up for the loss of direct access. Single port has taken away the triangulation from laparoscopy but MAGS technology may reintroduce this triangulation, although intra-abdominally, to make up for the deficiency. With minimal access surgery changing at a rapid pace, only longer follow-up and controlled randomised studies will tell if single-incision laparoscopy is a meaningful and lasting technique or a stepping stone towards a truly scarless intervention.[70]
